# InGaN micro-light-emitting diodes monolithically grown on Si: achieving ultra-stable operation through polarization and strain engineering

**DOI:** 10.1038/s41377-022-00985-4

**Published:** 2022-10-10

**Authors:** Yuanpeng Wu, Yixin Xiao, Ishtiaque Navid, Kai Sun, Yakshita Malhotra, Ping Wang, Ding Wang, Yuanxiang Xu, Ayush Pandey, Maddaka Reddeppa, Walter Shin, Jiangnan Liu, Jungwook Min, Zetian Mi

**Affiliations:** 1 Department of Electrical Engineering and Computer Science, University of Michigan, Ann Arbor, MI 48109 USA; 2 Department of Materials Science and Engineering, University of Michigan, Ann Arbor, MI 48109 USA

**Keywords:** Inorganic LEDs, Electronics, photonics and device physics

## Abstract

Micro or submicron scale light-emitting diodes (µLEDs) have been extensively studied recently as the next-generation display technology. It is desired that µLEDs exhibit high stability and efficiency, submicron pixel size, and potential monolithic integration with Si-based complementary metal-oxide-semiconductor (CMOS) electronics. Achieving such µLEDs, however, has remained a daunting challenge. The polar nature of III-nitrides causes severe wavelength/color instability with varying carrier concentrations in the active region. The etching-induced surface damages and poor material quality of high indium composition InGaN quantum wells (QWs) severely deteriorate the performance of µLEDs, particularly those emitting in the green/red wavelength. Here we report, for the first time, µLEDs grown directly on Si with submicron lateral dimensions. The µLEDs feature ultra-stable, bright green emission with negligible quantum-confined Stark effect (QCSE). Detailed elemental mapping and numerical calculations show that the QCSE is screened by introducing polarization doping in the active region, which consists of InGaN/AlGaN QWs surrounded by an AlGaN/GaN shell with a negative Al composition gradient along the *c*-axis. In comparison with conventional GaN barriers, AlGaN barriers are shown to effectively compensate for the tensile strain within the active region, which significantly reduces the strain distribution and results in enhanced indium incorporation without compromising the material quality. This study provides new insights and a viable path for the design, fabrication, and integration of high-performance µLEDs on Si for a broad range of applications in on-chip optical communication and emerging augmented reality/mixed reality devices, and so on.

## Abstract (web summary)

Micro-light-emitting diodes (µLED) with bright, ultra-stable green emissions are monolithically achieved on Si substrate. The quantum-confined Stark effect is screened by introducing polarization doping in the shell.

## Graphical abstract

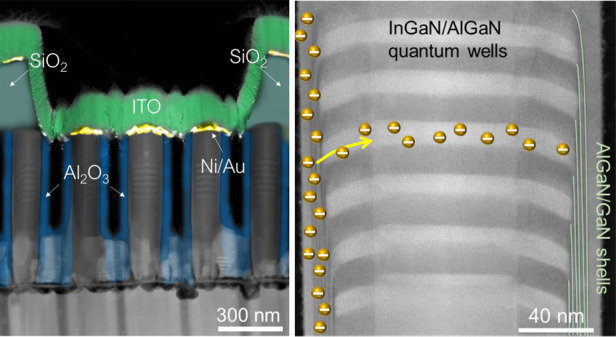

## Introduction

The past two decades have witnessed the solid-state lighting revolution powered by GaN-based broad area light-emitting diodes (LEDs), which generally have lateral dimensions on the order of millimeters. For the emerging revolution in augmented reality (AR)/mixed reality (MR), however, LEDs with dimensions as small as one micrometer, i.e., LEDs with a surface area approximately one million times smaller than conventional broad area devices, are in demand^1–12^. It is desired that the micro, or submicron scale LEDs (µLEDs) can exhibit highly stable emission, high efficiency and brightness, ultralow power consumption, and full-color emission, and can be monolithically grown on Si for integration with complementary metal-oxide-semiconductor (CMOS) electronics. To date, however, it has remained a daunting challenge to achieve such µLEDs, especially in the emission wavelength range of green/red.

Conventional InGaN quantum well (QW)-based LEDs suffer from wavelength/color instability due to the quantum-confined Stark effect (QCSE), which manifests itself as a severe wavelength blue-shift with increasing optical excitation or electrical injection^13–15^. Extensive efforts have been devoted to the development of nonpolar/semipolar III-nitride optoelectronics^16–20^. However, it remains elusive to achieve nonpolar/semipolar substrates with good quality and low cost. It was also shown that the emission wavelength instability issue can be mitigated through coupling the emissions of µLEDs into a microcavity, such as a photonic crystal cavity or Fabry-Pérot cavity^21,22^, wherein the dominant emission wavelength depends on the optical modes of the microcavity and is, therefore, less sensitive to the carrier density within the QWs. However, this method poses significant challenges in the device fabrication process and does not address the QCSE directly.

Other than the issues related to QCSE, the performance of green/red µLEDs remains limited since a relatively low growth temperature is generally required to achieve high indium composition, which significantly increases point defect density and impurity incorporation in the active region^23–25^. When the lateral dimensions of InGaN QW µLEDs are shrunk to below the micrometer scale, the external quantum efficiency often drops to well below 1%^26–28^, due to surface damage-induced nonradiative recombinations caused by top-down etching processes^28–32^. Moreover, there have been few studies of µLEDs monolithically directly grown on Si substrate^4,33,34^, due to the large lattice mismatch between GaN and Si.

In this study, we show that these conundrums can be addressed by adopting an AlGaN/GaN shell surrounding the active region with a negative Al composition gradient along the *c*-axis of InGaN nanowire µLEDs. The µLEDs are fabricated based on a N-polar nanowire array synthesized through a bottom-up selective area epitaxy (SAE) process. Detailed power-dependent photoluminescence (PL) measurements and current-dependent electroluminescence (EL) measurements show negligible shifts in the peak energy. Elemental mapping and theoretical calculations show that the QCSE is largely eliminated as a result of polarization doping. Strain distribution in InGaN QW is mapped using scanning transmission electron microscopy, which provides direct evidence that the use of AlGaN as the barrier can effectively reduce strain distribution in the active region and lead to enhanced indium incorporation. The devices have lateral dimensions below 1 µm and exhibit strong ultra-stable green emission. Moreover, the µLEDs are achieved on Si substrates instead of the commonly used sapphire substrate^35,36^, which reduces thermal accumulation within the device area and enables seamless integration with CMOS electronics. This work provides new insights and a viable approach for achieving high-performance µLEDs with highly stable operation on Si for their emerging applications in AR/MR, ultrahigh-resolution full-color displays, and other applications.

## Results

**Fig. 1:**
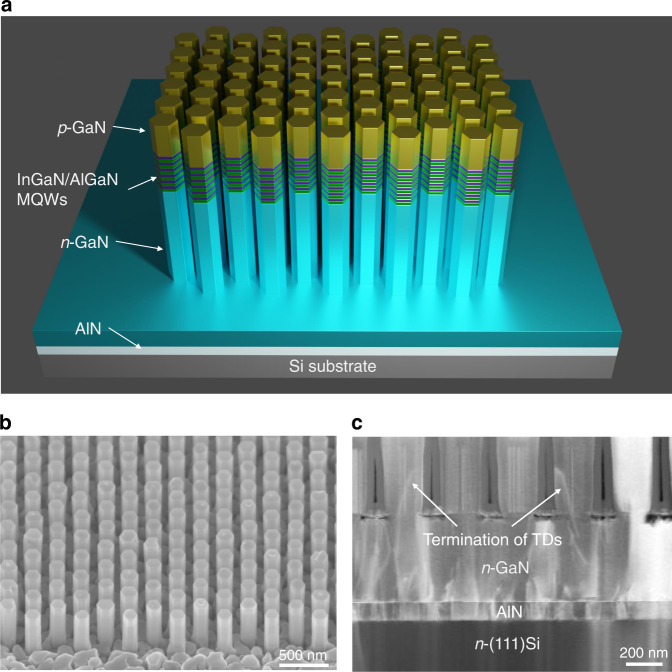
Schematic illustration of SAE nanowires on Si substrate and the filtering effect of nanowires on substrate dislocations. **a** Schematic of InGaN/AlGaN MQWs-in-nanowire heterostructure grown on Si substrate. **b** Bird’s view SEM image of the as-grown InGaN/AlGaN *p-i-n* nanowire arrays. **c** Cross-sectional STEM image of the nanowire on Si substrate showing the termination of TDs on the sidewall of *n*-GaN nanowire before reaching the active region

The nanowire µLED heterostructures are grown on a N-polar GaN/AlN buffer layer on Si wafer using plasma-assisted molecular beam epitaxy (PAMBE). The scanning electron microscopy (SEM) image of the MBE grown *n*-type N-polar GaN on Si template is shown in Fig. S1a in the Supplementary Information. Before performing SAE on the N-polar GaN on Si substrate, a thin Ti mask with nanoscale opening apertures is prepatterned on the substrate surface as shown in Fig. S1b. Detailed patterning processing can be found in our previous reports^37–39^. As schematically shown in Fig. 1a, each nanowire consists of ~450 nm Si-doped GaN, six periods of InGaN/AlGaN multiple QWs (MQWs), and ~170 nm Mg-doped GaN. Detailed growth parameters can be found in Methods. Figure 1b is a representative SEM image of the nanowire array grown by SAE on Si substrate. Each nanowire is enclosed by six equivalent *m*-planes on the sidewalls and a flat top *c*-plane, contrasting the pyramidal ($1\bar 10n$) top surfaces observed in metal-polar nanowires. The epitaxially grown nanowires inherit the lattice-polarity of the GaN/Si template, and the N-polarity is further confirmed by potassium hydroxide (KOH) etching as shown in Fig. S2, wherein the top surface is roughened with pyramidal islands. Despite the high dislocation density within the GaN epilayer, the majority (>85%) of the nanowires feature nearly perfect crystallinity, indicating a filtering effect of the nanowire structure on propagating dislocations^40^. The threading dislocation density (TDD) within the GaN film is measured to be ~3 × 10^10^ cm^−2^ ^41^. The TDs are observed to terminate on the sidewall of the nanowires, as shown in the cross-sectional scanning transmission electron microscopy (STEM) image (Fig. 1c), significantly reducing the defect density in the active region. This is exceptionally true for nanowires with diameters less than 150 nm, whereas larger diameter (over 200 nm) nanowires have a higher dislocation density, as shown in Fig. S3.

**Fig. 2:**
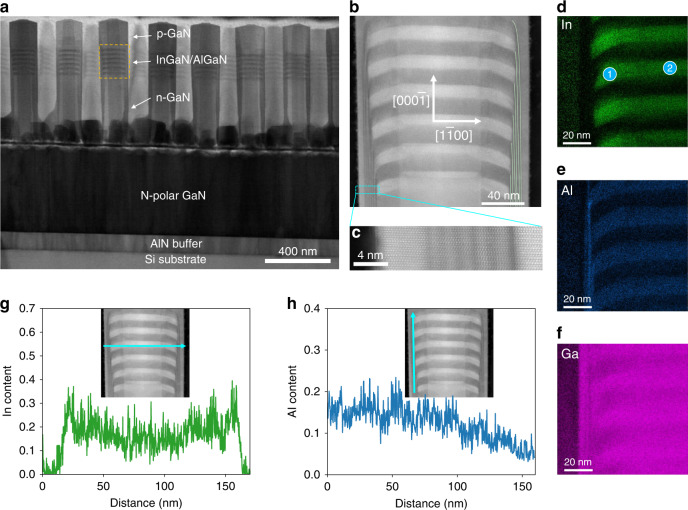
Structural characterizations of InGaN MQWs-in-nanowire heterostructure grown on Si substrate. **a** Low-magnification bright-field STEM image of InGaN/AlGaN nanowire heterostructure grown on GaN/AlN/Si substrate. **b** Magnified HAADF-STEM image of the orange-boxed region in **a**. The green curves indicate the AlGaN shells formed around the active region. **c** High-magnification HAADF image of the cyan-boxed region in **b**, wherein the six pairs of GaN/AlGaN superlattice on the sidewall are observed. **d**–**f** EDS element maps of the active region of InGaN/AlGaN MQWs. Indium composition point scans were performed at point 1 and 2 as indicated in **d**. **g** Indium element profile along the cyan arrow as shown in the inset. **h** Al element profile along the cyan arrow as shown in the inset

Detailed structural characterizations were performed on the as-grown InGaN/AlGaN MQWs-in-nanowire heterostructure. Figure 2a shows a low-magnification bright-field STEM image of the InGaN/AlGaN *p*-*i*-*n* heterostructure on the Si substrate, wherein individual layers of the heterostructure can be distinguished. The corresponding Si, Ga, and Al element maps collected by X-ray energy dispersive spectroscopy (EDS) clearly illustrate the SAE nanowires, GaN epi-film, AlN buffer, and the Si substrate as shown in Fig. S4. Figure 2b shows the high angle annular dark-field (HAADF) image of the active region, which consists of six pairs of InGaN/AlGaN QWs. The InGaN QWs show approximately uniform thicknesses (~12 nm) from QW to QW separated by AlGaN barriers along the *c*-axis. The edge of the QWs is found to be on the semipolar plane instead of the *c*-plane, indicating a partially faceted top surface during nanowire growth. The repeating growth of each AlGaN barrier introduces a shell structure around the active region, as indicated by the green lines in Fig. 2b, such that the bottom InGaN QW is surrounded by six periods of intensity-modulated shells (Fig. 2c) while the top InGaN QW is enclosed by one layer of the AlGaN shell. EDS elemental analyses (Fig. 2d–f) illustrate the distribution variations of different elements in the active region. The In-map and Al-map show the alternating growth of InGaN and AlGaN layers. One should note that negligible indium signals are detected from the shells. Therefore, the intensity modulation in the shell originates from an ultrathin GaN/AlGaN superlattice on the *m*-planes. The In-map exhibits stronger signals for InGaN incorporated on the semipolar plane compared to that on the *c*-plane, wherein the measured indium composition is ~30% (26%) at point 1 (2) of Fig. 2d. This is also confirmed by the EDS line scan along the horizontal cyan arrow in Fig. 2g, although one should note that the indium composition at the center of InGaN QW is underestimated due to a smaller overlap with the scan path. The Al composition in the AlGaN barrier is ~15%, as shown in EDS line scan results (Fig. S5). The EDS line scan results along the vertical cyan arrow in Fig. 2h show a negative Al composition gradient along the growth direction. The formation of such a unique structure can be attributed to the diffusion-controlled growth mechanism of III-nitride nanowire and the differences in incorporation efficiency on different crystalline planes^42–45^. More detailed explanations can be found in Section 6 of the Supplementary Information.

**Fig. 3:**
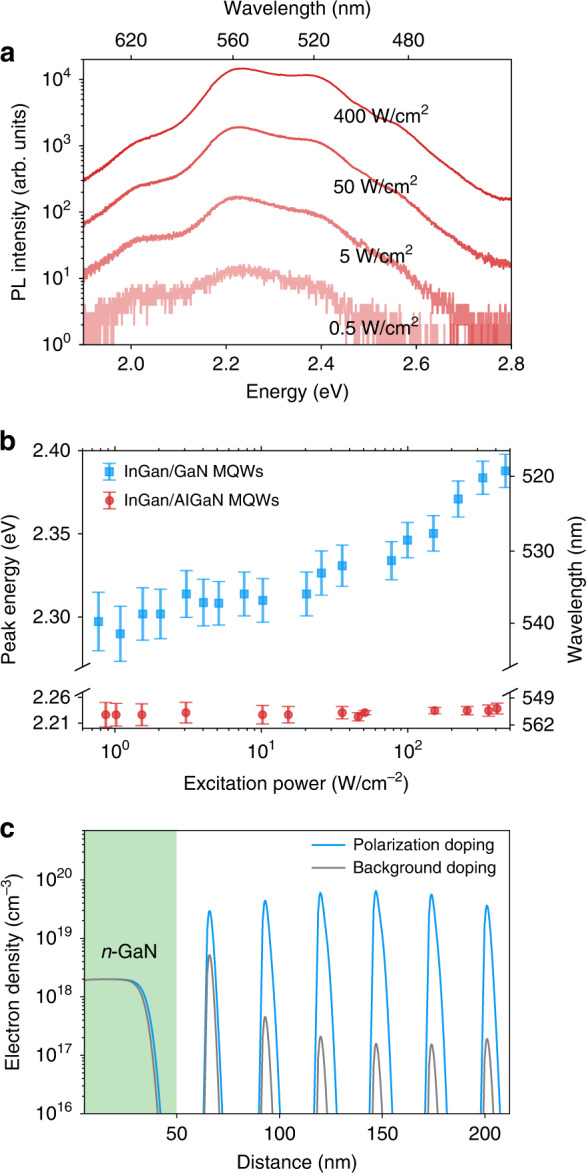
Comparisons of PL properties of InGaN/(Al)GaN MQWs and screening of QCSE through polarization doping. **a** Room-temperature power-dependent PL spectra of Sample A at 300 K with excitation power varied by ~3 orders of magnitude. **b** Emission peak energy vs. excitation power for Sample A (red circles) and Sample B (blue squares). **c** Calculated electron density distribution with the presence of polarization doping (blue curve)/background doping (gray curve). The green shaded region indicates the *n*-GaN region

Power-dependent PL properties of samples with AlGaN barrier (Sample A) and GaN barrier (Sample B) are investigated under resonant excitation with a 405 nm continuous wave (CW) laser at room temperature. The excitation power density is varied from ~0.5 to ~400 W·cm^−2^. Two dominant peaks can be identified from the spectra of Sample A, as shown in Fig. 3a. The dominant peak at 2.23 eV (556 nm) can be attributed to the InGaN QWs on the semipolar plane with a higher indium composition, whereas the peak around 2.36 eV (525 nm) can be attributed to the InGaN QWs on the *c*-plane with a lower indium composition as shown in Fig. 2d. The internal electrostatic field within the InGaN QWs of Sample A is calculated to be about 1.3 MV·cm^−1^ with spontaneous polarization and piezoelectric polarization considered. (Details can be found in Section 7 of Supplementary Information). The field of this strength, along with QW thickness of ~12 nm, is expected to cause severe separation of carrier wavefunctions and blue-shift in peak energy with increasing carrier density^46,47^. However, it can be seen from Fig. 3a and the red dots in Fig. 3b that over a wide excitation power density range, the sample exhibits almost constant peak energy with increasing excitation power, i.e., an ultra-stable emission. In addition, the screening of QCSE always comes along with a narrowing of the linewidth^48,49^. Here, the lack of this behavior in the excitation power dependence of the full-width-half-maximum (FWHM) confirms once more that the QCSE in the InGaN QWs of Sample A is negligible. The power-dependent PL spectra of Sample B can be found in Fig. S6, and the peak energy with excitation power is shown in Fig. 3b, wherein a blue-shift of ~100 meV can be observed over the studied excitation power range, because of the screening of QCSE within InGaN QWs.

The effect of Al incorporation on the carrier radiative recombination process can be related to the Al distribution in the active region. III-nitrides are polar materials, and the grading of (Al, Ga)N composition along the *c*-axis induces polarization charges in the bulk nanowire, which further results in accumulated free charge carriers with the opposite sign^50–52^. In this study, the Al content features a negative gradient along the $[000\bar 1]$ direction, which leads to a distribution of positive fixed charge in the shell region. In the presence of donor-type surface states and bulk shallow donors, the positive bound charges induce free electrons, which subsequently transfer to the InGaN QWs in the nanowire core region and thus screen the internal electrostatic field. The electron density distribution is then calculated by considering an epilayer heterostructure consisting of six pairs of 12 nm In_0.3_Ga_0.7_N QWs/15 nm Al_0.15_Ga_0.85_N barriers on a 50 nm thick *n*-GaN, similar to the experimental design of the InGaN/AlGaN heterostructure. An electron density of 3 × 10^17^ cm^−3^ is applied to the AlGaN barrier region, wherein the value is estimated based on the measured Al content change and induced polarization doping in Fig. 2h. The calculation is performed by solving the Schrödinger–Poisson equation iteratively. As shown in the blue curve in Fig. 3c, the electron densities within the InGaN QWs range from 3 × 10^19^ to 6 × 10^19^ cm^−3^ in the presence of induced free electrons, well above the reported Mott density of nitride QWs^50,53^. In contrast, the electron density drops below the degenerate doping when a uniform background doping of ~1 × 10^16^ cm^−3^ is adopted throughout the active region, further confirming that the absence of QCSE is due to the relocation of induced free electrons.

**Fig. 4:**
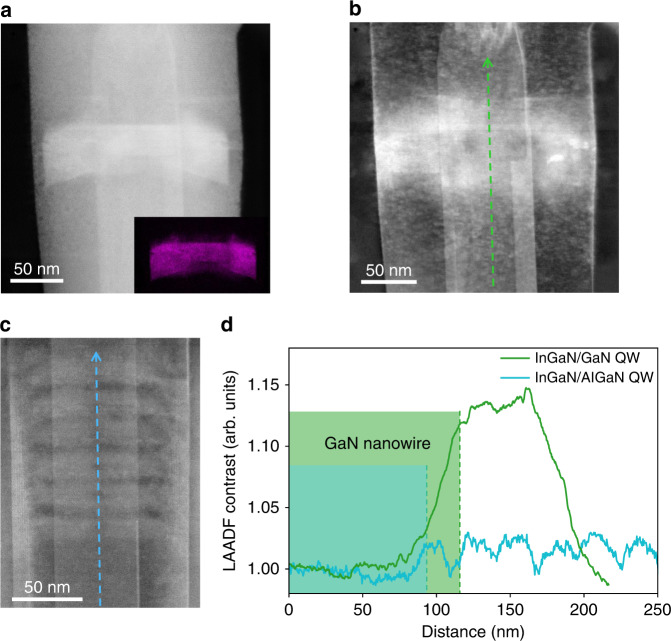
Strain mapping of InGaN/(Al)GaN MQWs-in-nanowire heterostructure. **a** HAADF image of the active region of Sample C. Inset, EDS map showing indium distribution in the active region of Sample C. **b** Corresponding LAADF image of **a**. **c** LAADF image of the active region and underlying GaN of Sample A. **d** LAADF line profile along the green dashed line in **b** and blue dashed line in **c**. The green (blue) shaded region indicates the GaN nanowire region in Sample C (Sample A)

Previous reports have suggested that AlGaN, due to a smaller lattice constant compared with GaN, can be used to compensate for the tensile strain caused by InGaN in the active region, which enhances the indium composition of InGaN QWs as a result of a smaller mismatch strain energy^54,55^. Although the strain field in heterostructures can be estimated by measuring lattice parameters using X-ray diffraction (XRD), the obtained values are susceptible to the dislocation distribution and limited to in-plane strain components rather than detailed three-dimensional strain distributions^54,56^. The strain distribution in InGaN/(Al)GaN MQWs is mapped, for the first time to our knowledge, using low angle annular dark-field (LAADF) STEM imaging. Sample C, which consists of a single InGaN QW in GaN nanowire, is grown for this study. Figure 4a shows the HAADF image of the active region of Sample C with its intensity proportional to the atomic number, Z. The high-Z cation sites dominate in the HAADF image, which is indium in this case, as confirmed by the In element map shown in the inset of Fig. 4a. The LAADF signals are collected at 20–50 mrad and are very sensitive to the effects, such as strain fields or phonons, that can cause the dechanneling of the incident electron beam^57^. This method has been used in measuring strain distributions in Si/Ge heterostructures^58^.

By matching the contrast variations in the simultaneously recorded HAADF and LAADF images (Fig. 4b), the strained regions can be identified. It is seen that the strained region diffused into about 20 nm into the bottom and top GaN barrier. Above this range, the strain field decays below the dechanneling detection limit. The bright column in the center of the nanowire is attributed to the thickness variation caused by focused ion beam (FIB) sampling. Figure 4c is the corresponding LAADF image of Fig. 2b, wherein the intensity change is significantly reduced across the active region compared with Fig. 4b. Figure 4d shows the LAADF line profile from the bottom GaN to the active region of Sample A and C where the signals are normalized to the respective bottom GaN to provide an estimate of the absolute strain field intensity in the active region. The observed peak contrast value of InGaN/GaN QW is much higher than that obtained from the InGaN/AlGaN QWs. The difference can be attributed to a reduced average lattice constant in the active region when the AlGaN barrier is adopted and hence a forced pseudomorphic growth of the entire MQW stack with significantly reduced strain relaxation. With reduced misfit strain energy, the indium composition increases, which is consistent with the longer emission wavelength observed from Sample A with the AlGaN barrier.

**Fig. 5:**
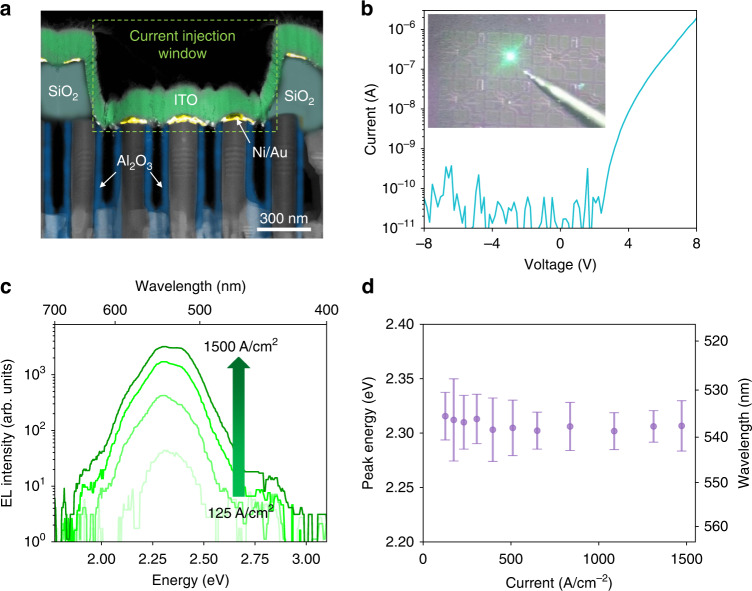
Performance characteristics of an InGaN µLED on Si. **a** Cross-sectional HAADF-STEM image of the as-fabricated InGaN/AlGaN μLED with an areal size of ~900 × 900 nm^2^. The current injection window is delineated by the green dashed box. Blue false color is applied to the Al_2_O_3_ passivation layer and the dark-cyan false color is assigned to the SiO_2_ insulation layer. Ni/Au layer is depicted by yellow false color and ITO is depicted by green false color. **b** *I-V* characteristics of as-fabricated InGaN/AlGaN nanowire μLEDs at room temperature, wherein negligible leakage current is observed at reverse bias. Inset: an optical image of the bright green emission measured from a µLED under an injection current of ~100 A·cm^−2^. **c** EL spectra under injection currents from 125 to 1500 A·cm^−2^ for an InGaN/AlGaN μLED. **d** Variation of peak position vs. injection current

µLED fabrication is performed by standard passivation, lithography, reactive ion etching, and metallization processes. Details about the fabrication process can be found in Methods. Devices with sizes of ~900 × 900 nm are fabricated. Figure 5a shows a cross-sectional HAADF-STEM image of a representative device with false color highlighting different materials. The blue false-colored region depicts the Al_2_O_3_ passivation layer on the sidewall of nanowires to prevent any leakage current caused by metal deposition. The top *p*-GaN of the nanowires is insulated by ~320 nm SiO_2_ (dark-cyan false-colored region) except for the nanowires located within the current injection window as indicated by the green dashed rectangle in Fig. 5a. Metallization of ~2.5 nm Ni/2.5 nm Au/180 nm ITO is used to form Ohmic contact with the top *p*-GaN of the nanowires after annealing at 550 °C in nitrogen ambient for 1 min. Each µLED consists of several individual nanowires with InGaN/AlGaN MQWs as the active region. The current-voltage (*I*-*V*) characteristics of the fabricated µLEDs show negligible leakage current under reverse bias, as shown in Fig. 5b, and the rectification ratio at ±8 V is over four orders of magnitude. The µLEDs are measured with a turn-on voltage of ~3 V. The inset of Fig. 5b shows an optical image of the bright green emission measured from a µLED under an injection current of ~100 A·cm^−2^. The current-dependent EL spectra are shown in Fig. 5c, wherein an ultra-stable emission with a dominant peak emission of ~2.3 eV (537 nm) was observed, as evidenced by the measured nearly invariant peak energy with increasing injection current (Fig. 5d). This is in direct contrast with previously reported green-emitting InGaN QW LEDs, wherein a blue-shift of 20–50 nm due to QCSE and color instability were widely observed^21,59,60^. With increasing injection current, the peak energy shifts from ~2.313 eV (536 nm) to 2.309 eV (537 nm). The slight red shift cannot be explained by the QCSE-related transitions reported in conventional InGaN/GaN MQWs. Instead, it can be explained by a combination of the current-induced heating effect and bandgap renormalization^61^. Significantly, in the wide InGaN QWs used in this study, the impact of Auger recombination can also be suppressed due to reduced carrier density within the InGaN QWs^62^, which can further promise reduced efficiency droop at elevated current density levels.

## Discussion

In conclusion, we show that the QCSE effect can be significantly suppressed by adopting AlGaN barriers in InGaN QWs µLEDs. This is achieved through a spontaneous formation of an AlGaN/GaN superlattice shell around the active region and a negative Al composition gradient from the bottom to the top of the active region. The free electrons induced by the polarization positive bond charges transfer into InGaN QWs and screen the QCSE. Moreover, the AlGaN quantum barrier can effectively compensate for the tensile strain caused by the InGaN QW in the GaN nanowire structure. The strain distribution within the active region is directly imaged and analyzed by LAADF-STEM. The strain compensation effect results in enhanced indium incorporation and thus a longer emission wavelength, and this is achieved without sacrificing the oscillation strength within the QWs. In addition, nanowire µLEDs consisting of the InGaN/AlGaN active region on Si substrate with submicron lateral dimensions have been demonstrated for the first time. The µLEDs feature ultra-stable green emissions with peak energies invariant with increasing injection currents in contrast to the blue shifts commonly measured from InGaN/GaN QWs because of QCSE. This work provides a new approach for designing the active region of high-performance multi-color µLEDs. Moreover, the monolithic integration of µLED on Si substrate is of great importance for next-generation display, optical communication, and other applications.

## Materials and methods

### Epitaxy

The growth of N-polar GaN epilayer on 2-inch Si(111) wafers was performed in a Veeco GENxplor system. The growth started with an unintentionally doped (UID) AlN buffer layer of ~100 nm and was followed by a Si-doped GaN layer of ~500 nm at a substrate temperature of 770 °C, nitrogen flow of 0.3 sccm, and plasma power of 350 W. After patterning and standard solvent cleaning, the wafer was loaded into a Veeco GEN II system for subsequent nanowire device structure growth. The Ti masked wafer was first nitrided at 400 °C for 10 min under a nitrogen plasma flow rate of 1 sccm. The *n*-GaN segment was grown at 690 °C, Ga beam equivalent pressure of 3.5 × 10^−7^ Torr, and nitrogen flow of 0.5 sccm. The estimated growth rate is ~3 nm·min^−1^. The active region was grown at a substrate temperature of 560 °C, Ga beam equivalent pressure (BEP) of 6 × 10^−8^ Torr, In BEP of 1 × 10^−7^ Torr, and Al BEP of 8 × 10^−9^ Torr. The nitrogen flow rate was increased to 0.7 sccm to enhance indium incorporation during active region growth. The *p*-GaN was grown at 690 °C for 30 min. Mg BEP of 8 × 10^−9^ Torr was used.

### Fabrication

The fabrication began with the atomic layer deposition (ALD) of Al_2_O_3_ to fill the air gap of the nanowire array. The top *p*-GaN of the individual nanowire was revealed by a fluorine-based reactive ion etching (RIE) process. Plasma-enhanced chemical vapor deposition of 320 nm SiO_2_ was performed as an insulation layer, followed by lithography and RIE etching to open the current injection window for each submicrometer LED. Metal stacks consisting of 2.5 nm Ni/2.5 nm Au/180 nm ITO were used as the *p*-metal contact. ITO was deposited via a sputtering process to ensure excellent coverage on the sidewall of the SiO_2_ insulation layer. Chlorine-based RIE process was used to etch down into the *n*-GaN. 20 nm Ti/80 nm Au was deposited for *n*-metal contact. The device was annealed in N_2_ ambient at 550 °C for 1 min.

### Characterizations

Structural properties of the samples were studied using a JEOL JEM-3100R05 analytical electron microscope with double Cs-correctors operated at 300 keV. EDS mapping was performed using Thermo Fisher Talos F200X analytical electron microscope. The *I-V* characteristics were measured using a Keithley 2400 voltage source meter. The PL and EL emissions were collected by an optical fiber and spectrally resolved by high-resolution spectrometers, and then detected by liquid nitrogen/thermal electrically cooled CCD cameras.

### Simulations

The electron densities were simulated by solving one-dimensional Schrödinger–Poisson equations via BandEng. The spontaneous polarization, piezoelectric, elastic, and lattice constants of Al_x_Ga_1−x_N and In_x_Ga_1-x_N used in the simulation were based on previously reported values and summarized in Table S1 in the Supplementary Information.

## Supplementary information

Supplementary Information

## Data Availability

The data that support the findings of this study are available from the corresponding authors upon request.
