# Supplementary material for: InGaN micro-light-emitting diodes monolithically grown on Si: achieving ultra-stable operation through polarization and strain engineering
Source: Light Sci Appl. 2022 Oct 10;11:294. doi: 10.1038/s41377-022-00985-4 (PMC9550839; doi:10.1038/s41377-022-00985-4)
Supplement: Supplementary file 1 — Supplementary Information [file 41377_2022_985_MOESM1_ESM.docx]

Supplementary Information for

InGaN Micro-light-emitting Diodes Monolithically Grown on Si: Achieving Ultra-stable Operation through Polarization and Strain Engineering

Yuanpeng Wu^1^, Yixin Xiao^1^, Ishtiaque Navid^1^, Kai Sun^2^, Yakshita Malhotra^1^, Ping Wang^1^, Ding Wang^1^, Yuanxiang Xu^1^, Ayush Pandey^1^, Reddeppa Maddaka^1^, Walter Shin^1^, Jiangnan Liu^1^, Jungwook Min^1^, Zetian Mi^1,*^

*^1)^Department of Electrical Engineering and Computer Science, University of Michigan, Ann Arbor, MI 48109, USA*

*^2)^Department of Materials Science and Engineering, University of Michigan, Ann Arbor, MI 48109, USA*

*^*)^Corresponding author:* [*ztmi@umich.edu*](mailto:ztmi@umich.edu)

**Section 1. Surface morphologies of the N-polar GaN on Si with and without Ti patterning**

A 10 nm thick Ti mask with nanoscale opening apertures is fabricated on the surface of the N-polar GaN on Si template before selective area epitaxy (SAE) of nanowire structure. As can be seen, some of the opening apertures overlap with the pits on the surface. During SAE of nanowires, the initial nucleation and subsequent elongation of nanowires occur exclusively within the opening aperture. Significantly reduced defect density in the as-grown nanowires was observed when the opening aperture diameter was less than 150 nm.

**
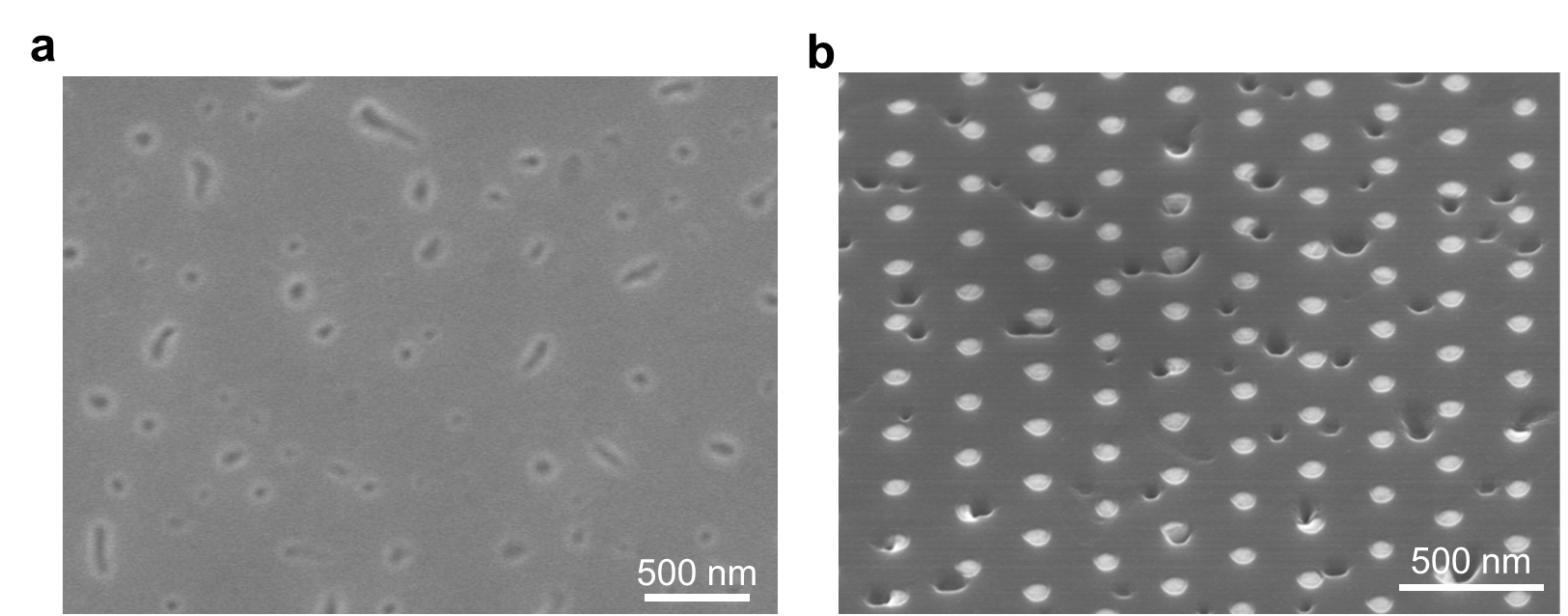
**

**Figure S1.** (a) Top view SEM image of the as-grown N-polar GaN on Si substrate. (b) Bird’s view SEM image of N-polar GaN patterned by a Ti mask with nanoscale opening apertures.

**Section 2. Confirmation of N-polarity of the SAE nanowires through chemical treatment**

The lattice-polarity of the SAE nanowire was examined by diluted potassium hydroxide (KOH) solution treatments. The observation of roughened top surface and pyramidal islands confirmed that the SAE nanowires inherit the N-polarity from the GaN buffer layer. The wet-etching process was kept short (20 s) to avoid a complete etching of the entire nanowire structure.

**
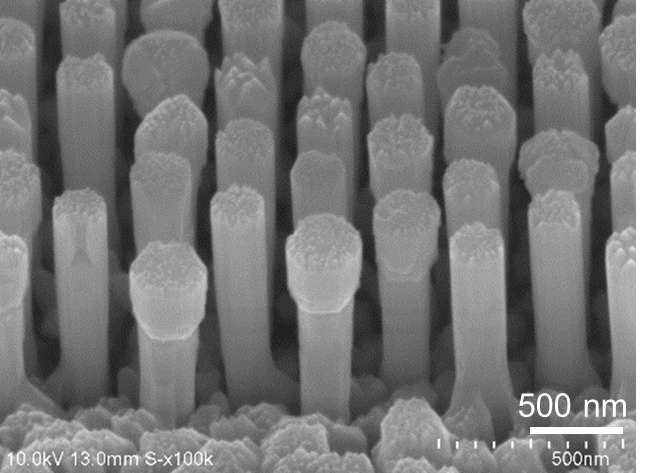
**

**Figure S2.** Bird’s view SEM image of nanowires etched in 10 w% KOH solution at 40 ºC for 20 s.

**Section 3. Reduced filtering effect on substrate dislocations of SAE nanowires with larger diameter**

With increased diameters of the SAE nanowires, the filtering effect on substrate dislocations reduces. As shown in Fig. S3, the majority of the SAE nanowires have observable structural defects on either the top surface or the sidewalls, in direct contrast to the morphologies of small diameter nanowires in Fig. 1b, wherein most of the nanowires feature nearly perfect structural quality.

**
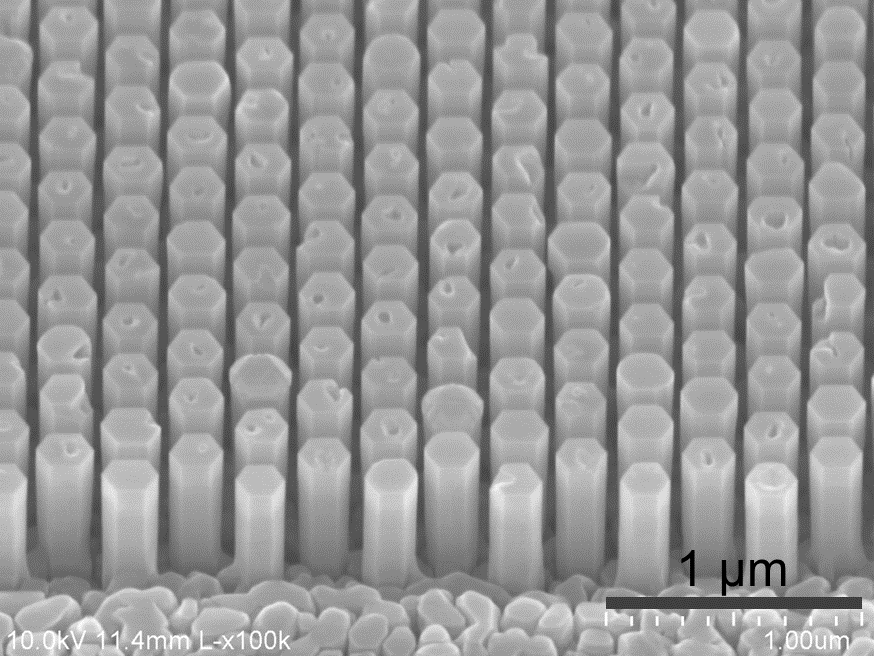
**

**Figure S3**. Bird’s view SEM image of nanowires with diameters of 210 nm.

**Section 4. EDS mapping of the SAE nanowire on GaN/AlN/Si substrate**

**
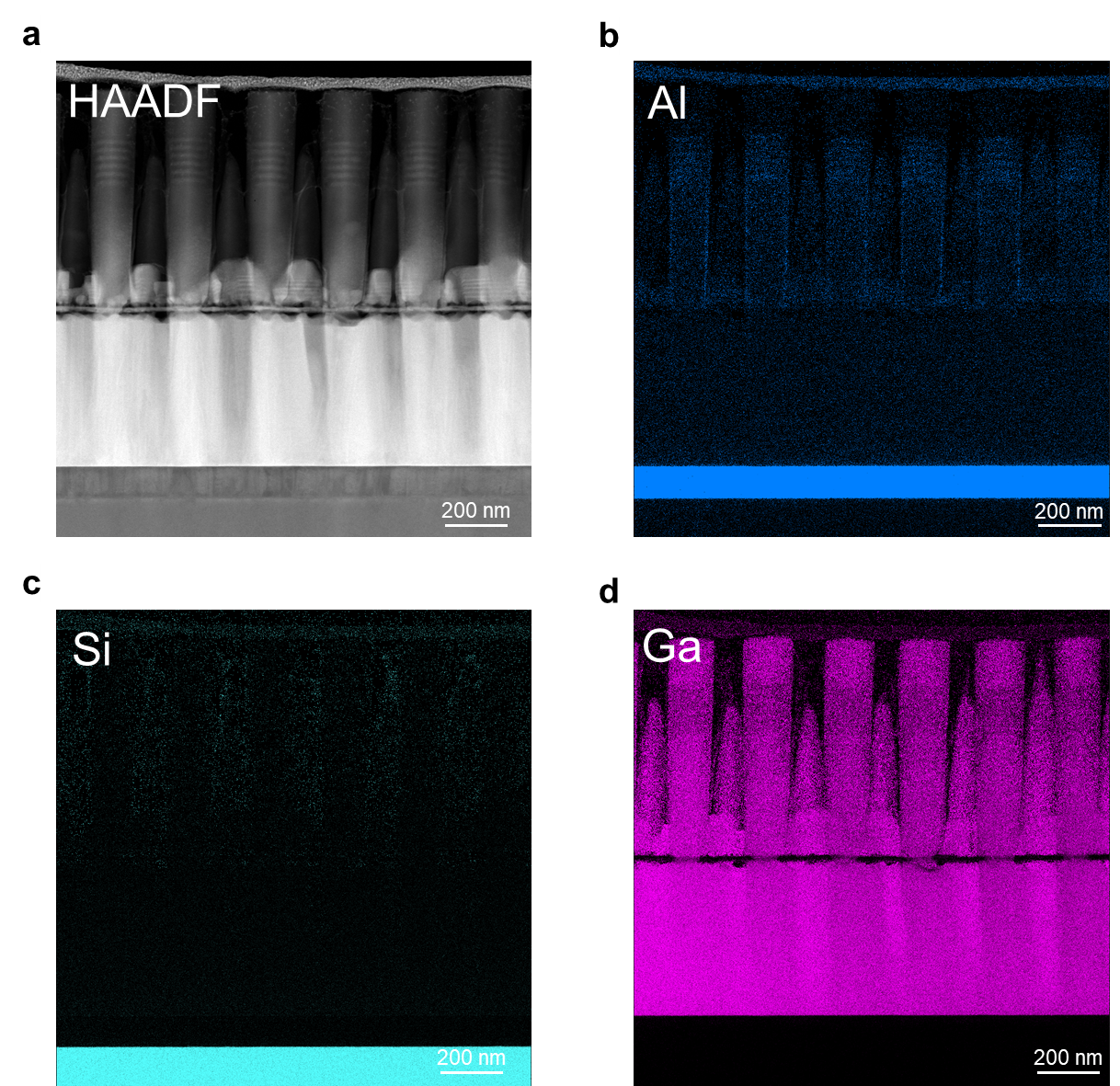
**

**Figure S4**. (a) Low magnification HAADF image of the nanowire on Si structure. (b-d) EDS element maps of Al, Si and Ga in the structure.

**Section 5. EDS Al line scan of AlGaN barrier**

**
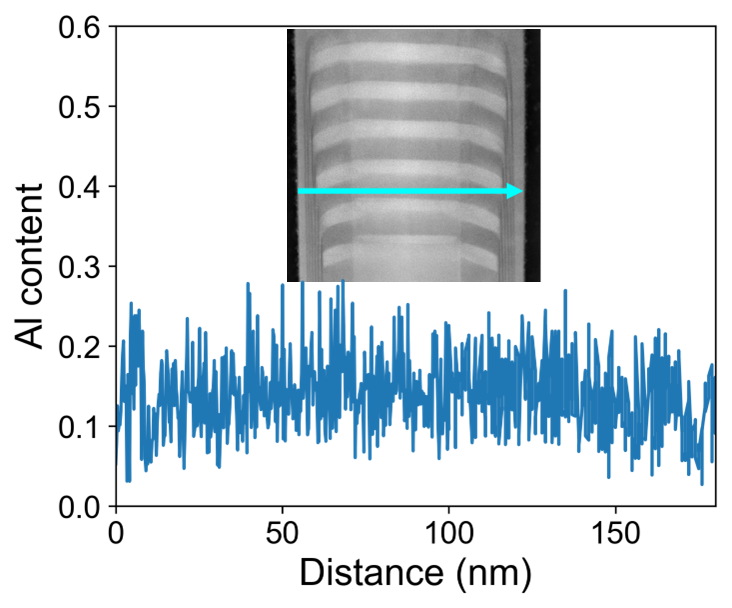
**

**Figure S5**. EDS Al line scan in the AlGaN barrier layers.

**Section 6. Discussion on the formation mechanism of GaN/AlGaN shell**

During the alternating growth of InGaN QW and AlGaN barriers of the active region, InGaN/AlGaN heterostructure is formed along the *c*-axis and GaN/AlGaN shell is formed on the sidewalls. The formation of the GaN/AlGaN shell can be mainly attributed to three factors. First, the diffusion lengths (*L*) of In, Ga and Al adatoms have the relation that *L*_In_>*L*_Ga_>*L*_Al_ and most In adatoms can efficiently migrate to the top surface and contribute to epitaxy of InGaN QW along the *c*-axis[1]. Meanwhile, the sidewall is dominated by Ga and Al surface adatoms, which leads to In deficiency and the formation of AlGaN/GaN shell. Second, the incorporation efficiency of indium is lower on the *m*-plane (sidewall) compared to that on *c*-plane (top facet)[2], which also contributes to the negligible indium composition on the sidewalls. Third, the growth temperature was relatively high such that any indium adatoms on the surface or near-surface region of the nanowires were eventually desorbed from the surface, leading to the absence of indium in the shell.

**Section 7. Calculation of electric field within InGaN QW**

Depending on the AlGaN barrier (*L*_b_) and the InGaN well thickness (*L*_w_), the total electric field within the InGaN QW can be expressed as [3],

$$F_{w}=\frac{L_{b}(P_{\mathrm{SP}}^{b}+P_{\mathrm{PZ}}^{b}-P_{\mathrm{SP}}^{w}-P_{\mathrm{PZ}}^{w})}{\varepsilon_{0}({L_{b}\varepsilon}_{w}+{L_{w}\varepsilon}_{b})}$$

where superscripts b and w represent the barrier and well, and $P_{\mathrm{SP}}$, $\varepsilon$ and *L* are the spontaneous polarization constant, static dielectric constant and the layer thickness, respectively. The strain-induced piezoelectric polarization in the QW $P_{\mathrm{PZ}}$ is given by [3],

$$P_{\mathrm{PZ}}=2\frac{a-a_{0}}{a_{0}}(e_{31}-e_{33}\frac{C_{13}}{C_{33}})$$

where $a$ and $a_{0}$ are the lattice constants of the overlayer and underlayer, respectively, $e_{31}$ and $e_{33}$ are the piezoelectric constants, $C_{13}$ and $C_{33}$ are the elastic constants. $a$, $a_{0}$, $e_{31}$, $e_{33}$, $C_{13}$ and $C_{33}$ of In_x_Ga_1-x_N and Al_x_Ga_1-x_N are estimated using Vegard’s law [4].

**Section 8. The power-dependent PL spectra of InGaN/GaN MQWs-in-nanowire heterostructure**

Power-dependent PL measurements were performed on InGaN/GaN MQWs-in-nanowire heterostructure (Sample B) with excitation power varied over three orders of magnitude.


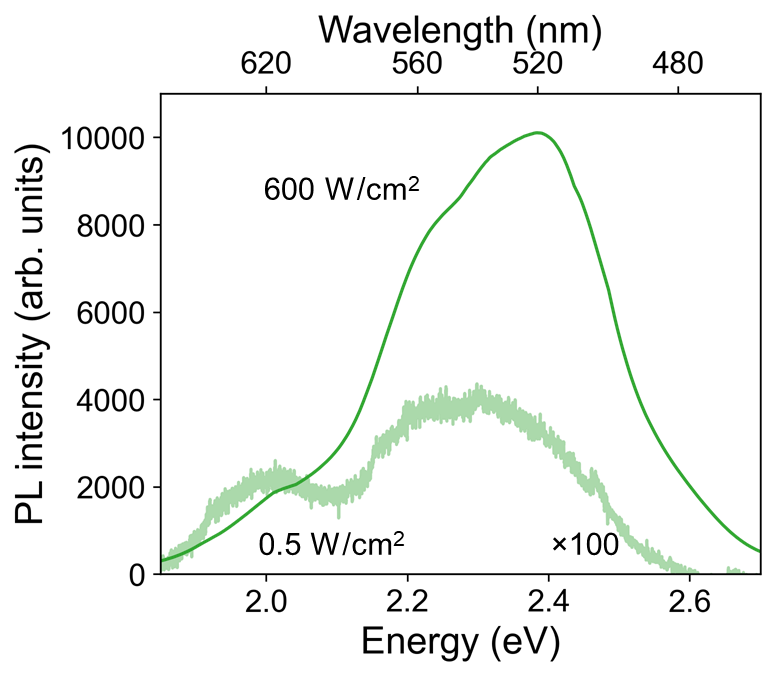


**Figure S6**. The power-dependent PL spectra of Sample B.

**Section 9.** Table S1: Spontaneous polarization, elastic and piezoelectric constants of Al_x_Ga_1-x_N and In_x_Ga_1-x_N used for electron density calculation.

|  | **Al_x_Ga_1-x_N** | **In_x_Ga_1-x_N** |
| --- | --- | --- |
| $P_{\mathrm{SP}}$ (C/m^2^) | -0.029+0.052$x$ [5] | -0.029-0.003$x$ [6] |
| $C_{13}$ (GPa) | 103+5$x$ [4] | 103-11$x$ [7] |
| $C_{33}$ (GPa) | 405+3$x$ [4] | 405-181$x$ [7] |
| $e_{13}$ (C/m^2^) | -0.46-0.14$x$ [5] | -0.46-0.08$x$ [7] |
| $e_{33}$ (C/m^2^) | 0.78+0.68$x$ [5] | 0.78+0.24$x$ [7] |

**References:**

1. Cheng, S. *et al*. Nanoscale Structural and Emission Properties within “Russian Doll”‐Type InGaN/AlGaN Quantum Wells. *Advanced Optical Materials* **8**, 2000481 (2020).

2. Sun, J. *et al*. In surface segregation in M-plane (In,Ga)N/GaN multiple quantum well structures. *Appl. Phys. Lett.* **83**, 5178-5180 (2003).

3. Park, S.-H. *et al*. Spontaneous polarization effects in wurtzite GaN/AlGaN quantum wells and comparison with experiment. *Applied Physics Letters* **76**, 1981-1983 (2000).

4. Ambacher, O. et al. Polarization induced interface and electron sheet charges of pseudomorphic ScAlN/GaN, GaAlN/GaN, InAlN/GaN, and InAlN/InN heterostructures. *J. Appl. Phys.* **129**, 204501 (2021).

5. Bernardini, F. *et al*. Spontaneous polarization and piezoelectric constants of III-V nitrides. *Physical Review B* **56**, R10024-R10027 (1997).

6. Belghouthi, R. *et al*. Modeling of polarization charge in N-face InGaN/GaN MQW solar cells. *Mater. Sci. Semicond. Process.* **40**, 424-428 (2015).

7. Routray, S.R. *et al*. Effect of degree of strain relaxation on polarization charges of GaN/InGaN/GaN hexagonal and triangular nanowire solar cells. *Solid·State Electron.* **159**, 142-149 (2019).
